# Early Identification of Severe COVID-19 Cases and the Need for ICU Care Based on Clinical and Laboratory Risk Factors

**DOI:** 10.7759/cureus.80611

**Published:** 2025-03-15

**Authors:** Jawad Mahmood, Muhammad Izhar Ul Haque, Maria Gul, Aliya Ayub, Fawwad A Ansari, Wiqas Ahmad

**Affiliations:** 1 Gastroenterology and Hepatology, Hayatabad Medical Complex, Peshawar, PAK; 2 Department of Comparative Biomedical Sciences, College of Veterinary Medicine, University of Georgia, Athens, USA; 3 Gynecology, Ayub Teaching Hospital, Abbottabad, PAK; 4 Epidemiology and Public Health, Institute of Public Health and Social Sciences, Khyber Medical University, Peshawar, PAK; 5 Internal Medicine, Piedmont Athens Regional Medical Center, Athens, USA

**Keywords:** covid-19, intensive care units (icus), pulmonary critical care, sars-cov-2, screening

## Abstract

Background and objective

Treatment in ICUs became extremely difficult due to the growing number of coronavirus disease 2019 (COVID-19) patients at the height of the pandemic. Consequently, prompt patient triage depends on the early categorization of severe cases in such scenarios. This study aimed to provide an evidence-based strategy to ensure the best use of resources by triaging patients based on objective risk factors.

Methods

This retrospective observational study comprised 500 inpatients (>age 18 years) who were hospitalized between March 20 and April 19, 2020, at the Khyber Teaching Hospital (KTH) and Hayatabad Medical Complex (HMC) in Peshawar, Pakistan. The clinical, laboratory, and radiological parameters were assessed. Real-time polymerase chain reaction (RT-PCR) findings were used to confirm the diagnosis of COVID-19.

Results

A total of 19 potential clinical and laboratory risk factors associated with ICU admissions were identified. At least one comorbidity among chronic lung disease, cardiovascular disease (CVD), and diabetes was the factor with the strongest association with ICU admission with a univariable odds ratio (OR) of over 27, followed by renal disease and other COVID-19 sequelae such as diarrhea, respiratory rate (>24 breaths/minute), and positive RT-PCR (vs. negative) with an univariable OR between 9 and 15. Furthermore, a multivariate logistic regression model was further developed with five risk factors, including comorbidity, presence of chronic lung disease, presence of diabetes, and RT-PCR (positive vs. negative), male sex (vs. female), and older age (65.0-80.5 years), suggesting a good fit of the model to the data shown by the area under the receiver operator characteristic curve (AUC) of 0.943 (95% CI: 0.917, 0.969). Additionally, a chest CT scan showed the typical COVID-19 pneumonia with pulmonary involvement of 30-40%, which was further evaluated by the COVID-19 Reporting and Data System (CO-RADS). The typical COVID-19 pneumonia was on a scale of four (15/25) or five (19/25) lung lesions.

Conclusions

Based on our findings, this approach could be used to screen the severe cases of COVID-19 patients and help them to be treated in ICUs on time while preventing others from unnecessarily using ICUs in the setting of limited medical resources, such as the outbreak of a pandemic.

## Introduction

As the number of patients affected by coronavirus disease 2019 (COVID-19), caused by severe acute respiratory syndrome coronavirus 2 (SARS‑CoV‑2) expanded significantly at the height of the pandemic, treatment in ICUs around the world imposed a major financial burden and was linked to facility and resource constraints, especially in developing nations where medical resources are limited [1]. In light of this, the timely identification of severe forms of COVID-19 cases is key for prioritizing and determining the treatment needs of the patients [2]. Resource allocation, particularly the management of ICU beds and ventilators, has been a significant challenge [3]. Hence, there is an urgent need to develop effective ways to facilitate early detection and triage of severe COVID-19 cases to optimize resource utilization and enhance patient outcomes [4].

A total of 1,581,936 confirmed cases of COVID-19 and 30,664 deaths were reported in Pakistan by April 13, 2024 [5]. Early identification of patients in need of ICU treatment and adoption of preventive measures are essential to alleviate the healthcare burden [6]. In the context of the global COVID-19 pandemic, this study attempted to establish an effective technique to identify severe cases early, particularly in resource-constrained nations like Pakistan, where ensuring prompt medical care faces several obstacles. Understanding the associations between clinical, hematological, biochemical, and radiological results will help doctors manage COVID-19 patients more efficiently, guaranteeing optimal use of medical resources and enhancing patient outcomes in the face of medical overpopulation.

## Materials and methods

Study design and participants

In this retrospective observational study, 500 adult inpatients (≥18 years) were recruited using convenient sampling from two teaching hospitals [Khyber Teaching Hospital (KTH) and Hayatabad Medical Complex (HMC) - both affiliated with Khyber Medical University (KMU) in Peshawar, Pakistan] from March 20 to April 19, 2020. These patients were tested for COVID-19 as per WHO guidelines and received regular in-patient care or intensive care based on the severity of the disease.

Once a clinically suspected case was identified, an investigation team, comprising resident physicians, conducted a detailed clinical assessment to gather primary information on demographic data such as clinical signs and the presence of fever and cough, from the patients. Data regarding seven common comorbidities, including the presence of chronic lung disease, diabetes, cardiovascular disease (CVD), cerebrovascular accident, renal disease, obesity, and a history of hemodialysis, were collected from the existing medical records.

Case definition and testing

A confirmed case was defined as clinical specimens testing positive by RT assay. 

Chest CT scans

On the day of patients' admission, the lungs' image status was assessed using chest CT scanning (Aestone, Toshiba, Japan). Each patient that was chosen for a chest CT scan was given a score (1-5) based on the COVID-19 Reporting and Data System (CO-RADS). The scores were assigned for each patient as follows: CO-RADS-1 (normal), CO-RADS-2 (abnormalities consistent with infections other than COVID-19), CO-RADS-3 (unclear whether COVID-19 is present), CO-RADS-4 (abnormalities suspicious for COVID-19), CO-RADS-5 (typical COVID-19), and CO-RADS-6 (positive RT-PCR). The degree of parenchymal involvement in each lobe was scored using the CT severity scoring systems (CTSS), yielding a total score of 25. Each lobe received a score between 1 and 5. The senior, experienced radiologists used the CTSS and CO-RADS to reconstruct and evaluate the CT scan pictures. [7]

Blood test

For hematology, 5 mL blood samples with the anticoagulant diamine tetra acetic acid (EDTA K3) were collected from all patients and subjected to a routine blood examination to measure the cell counts (total leukocytes, differential leucocytes, lymphocytes, neutrophils, basophils, platelets), hemoglobin values and hematocrit. For serum biochemical parameters, the serum was separated from the blood without the anticoagulant by centrifugation at 11,000 g for 10 minutes. Serum biochemical parameters including C-reactive protein (CRP), lactate dehydrogenase (LDH), creatinine kinase (CK), ferritin, alanine aminotransferase (ALT), aspartate aminotransferase (AST), alanine transaminase (ACT), and total protein levels were measured by using an automatic chemistry analyzer (Roche Cobas C111 Chemistry Analyzer, Northwest Lab Source, LLC, East Sumner, WA).

Statistical analysis

The R-statistical program (R statistical package version 4.0.2, R Development Core Team 2020) was used for the subsequent data analysis once all data were entered into a Microsoft Excel spreadsheet. The median [interquartile range (IQR)] and n (%), respectively, were used to represent continuous and categorical variables. When applicable, the differences between patients admitted to the ICU and those in general isolation wards (isolation wards) were compared using the Mann-Whitney U test, Pearson's Chi-square test, or Fisher's exact test. Univariable and multivariable mixed-effects logistic regression models were created to examine the risk factors linked to ICU admission. Hospitals were included as a random effect in the models to account for the possibility of patient clustering among various hospitals.

Multivariable logistic regression analysis was used to evaluate variables that showed a p-value of less than 0.20 in the univariable studies. There was no collinearity between the variables, according to the variance inflation factors of all the included variables, which ranged from 1.03 to 1.16. To control for confounding variables, a backward stepwise procedure was used to develop a multivariable logistic regression model. The final model included variables with p-values less than 0.05. AIC (Akaike's Information Criteria) and the Hosmer-Lemeshow goodness-of-fit test were used to perform goodness-of-fit tests to assess the models. The model with the least AIC value was considered the most appropriate. Using the R package 'pROC', a receiver operating characteristic (ROC) curve was developed to demonstrate the model's predicted accuracy. (Robin et al., 2011). Odds ratios (OR) and 95% CI were also used to determine the degree of relationship between variables.

## Results

Baseline characteristics of patients infected with SARS-CoV-2

Clinical signs and SARS-CoV-2 testing results with RT-PCR of the 500 patients are summarized in Table 1. The median age of the patients was 49 years (IQR: 41.0-63.3) and most (73.4%) were male. Nine patients (18%) were admitted to ICU and these had a significantly higher age than those not admitted to the ICU (median age: 73 vs. 47 years, p<0.001). Male patients were slightly more likely to be admitted to ICU than females, although not significantly (p=0.068).

**Table 1:** Comparison of demographics and clinical manifestations between ICU and non-ICU patients ICU: intensive care unit

Demographics and clinical manifestations				
Parameter	Total (n=500)	ICU patients (n=90)	Non-ICU patients (n=410)	P-value
Age, years	49.0 (41.0-63.3)	73.0 (65.0-80.5)	47.0 (38.0-56.0)	<0.001
Sex				
Male	367 (73.4%)	73 (81.1%)	294 (71.7%)	0.068
Female	133 (26.6%)	17 (18.9%)	116 (28.3%)	
Presence of pneumonia-related manifestations				
Respiratory rate (>24 breaths/minute)	225 (45.0%)	77 (85.6%)	148 (36.1%)	<0.001
Diarrhea	10 (2.0%)	7 (7.8%)	3 (0.7%)	<0.001
Myalgia	335 (67.0%)	71 (78.9%)	264 (64.4%)	0.008
Cough	230 (46.0%)	48 (53.3%)	182 (44.4%)	0.123
Fever (≥37.3 ℃)	395 (79.0%)	66 (73.3%)	329 (80.2%)	0.145
Neurological signs	35 (7.0%)	3 (3.3%)	32 (7.8%)	0.172
Rhinorrhea	65 (13.0%)	15 (13.0%)	50 (12.2%)	0.253
Excess sputum production	110 (22.0%)	23 (25.6%)	87 (21.2%)	0.369
Systolic blood pressure (<90 mmHg)	110 (22.0%)	23 (25.6%)	87 (21.2%)	0.369
Presence of comorbidity	66 (13.2%)	51 (56.7%)	15 (3.7%)	<0.001
Chronic lung disease	48 (9.6%)	39 (43.3%)	9 (2.2%)	<0.001
Diabetes	23 (4.6%)	19 (21.1%)	4 (1.0%)	<0.001
Cardiovascular disease	7 (1.4%)	6 (6.7%)	1 (0.2%)	<0.001
Renal disease	4 (0.8%)	3 (3.3%)	1 (0.2%)	0.02
Obesity	4 (0.8%)	4 (4.4%)	0 (0.0%)	-
Cerebrovascular accident	2 (0.4%)	2 (2.2%)	0 (0.0%)	-
Hemodialysis history	1 (0.2%)	1 (1.1%)	0 (0.0%)	-

The most common clinical symptoms were fever (79.0%), myalgia (67.0%), and cough (46.0%), followed by excess sputum production (22.0%), rhinorrhea (13.0%), neurological signs (7.0%) and diarrhea (2.0%). However, high respiratory rate (>24 breaths per minute) (normal: 12-20 breaths/minute) (p<0.001), diarrhea (p<0.001), and myalgia (p=0.008) occurred more frequently in the patients admitted to ICU than those in the isolation wards (Table 1).

Seven comorbidities were assessed for their association with ICU admission. Although comorbidities were present in only 13.2% of the patients, there was a significant difference in the frequency of comorbidities between ICU and isolation ward patients (p<0.001), with chronic lung disease being the most common comorbidity (9.6%), followed by diabetes (4.6%) and CVD (1.4%). The remaining comorbidities all accounted for less than 1% (Table 1). Although treatment was given based on corresponding symptoms, it was not surprising that the mortality of ICU patients (12.2%, 95% CI: 6.3, 20.8) was significantly higher than that of isolation wards (1.0%, 95% CI: 0.3, 2.5) with an OR of 14.1 (95% CI: 4.4, 45.5; p<0.001).

Laboratory findings

Of note, 350 patients (70.0%, 95% CI: 65.8, 74.0) tested positive by RT-PCR, with significantly more patients in the ICU (94.4%, 95% CI: 87.5, 98.2) testing positive than those in isolation units (64.6%, 95% CI: 59.8, 69.3, p<0.001). Regarding serum biochemical parameters, over 92% of patients suffered from elevated LDH, ferritin, and CRP, followed by elevated levels of ALT, urea, AST, and creatinine kinase (CK) (>1.1 mg/dL). The decline in serum total protein levels was found in 63.0% (95% CI: 58.6%, 67.2%) of the patients; while the proportion of hyponatremia (<135 mEq/L), hypochloremia (<98 mEq/L), and hypokalemia (<3.5 mEq/L) in the patients were 29.8% (95% CI: 25.8%, 34.0%), 24.0% (95% CI: 20.3%, 28.0%), and 16.0% (95% CI: 12.9%, 19.5%), respectively (Table 2). When comparing the patients in ICU with those in isolation wards, only five serum biochemical parameters showed a statistical difference between the two groups (significantly elevated CK, hypernatremia, hypokalemia, urea, and ALT; all p<0.05; Table 2). Approximately 90% of the patients had abnormal hematological results, although there was no significant difference between ICU and isolation ward patients in this regard (Table 2).

**Table 2:** Differences in laboratory findings between ICU and non-ICU patients ICU: intensive care unit

Parameter	Total (n=500)	ICU patients (n=90)	Non-ICU patients (n=410)	P-value
Positive real-time polymerase chain reaction (RT-PCR)	350 (70.0%)	85 (94.4%)	265 (64.6%)	<0.001
Elevated creatinine kinase (CK, >1.1 mg/dL)	231 (46.2%)	26 (28.9%)	205 (50.0%)	<0.001
Hyponatraemia (<135 mEq/L)	149 (29.8%)	14 (15.6%)	135 (32.9%)	0.001
Elevated alanine aminotransferase (ALT, >33 U/L)	360 (72.0%)	76 (84.4%)	284 (69.3%)	0.004
Elevated urea (>48.5 mg/dL)	270 (54.0%)	59 (65.6%)	211 (51.5%)	0.015
Hypokalemia (<3.5 mEq/L)	80 (16.0%)	8 (8.9%)	72 (17.6%)	0.042
Hypochloremia (<98 mEq/L)	120 (24.0%)	19 (21.1%)	101 (24.6%)	0.479
Elevated lactate dehydrogenase (LDH, >280 U/L)	480 (96.0%)	88 (97.8%)	392 (95.6%)	0.552
Elevated ferritin (>400 ng/mL)	495 (99.0%)	89 (98.9%)	406 (99.0%)	1
C-reactive protein (>5.0 mg/dL)	460 (92.0%)	81 (90.0%)	379 (92.4%)	0.44
Elevated aspartate aminotransferase (AST, >375 U/L)	260 (52.0%)	47 (52.2%)	213 (52.0%)	0.963
Decreased total protein (<60 mg/dL)	315 (63.0%)	60 (66.7%)	255 (62.2%)	0.426
Decreased platelet count (<150 k cells/µL)	470 (94.0%)	86 (95.6%)	384 (93.4%)	0.628
Elevated white blood cell count (>10 k cells/µL)	460 (92.0%)	84 (93.3%)	376 (91.7%)	0.607
Decreased red blood cell count (<4.5 × 1012 cells/uL)	439 (87.8%)	80 (88.9%)	359 (87.6%)	0.727
Decreased hemoglobin (<13 g/dl)	450 (90.0%)	82 (91.1%)	368 (89.8%)	0.698
Elevated neutrophils (>75%)	450 (90.0%)	81 (90.0%)	369 (90.0%)	1
Decreased lymphocytes (<20%)	465 (93.0%)	90 (100.0%)	375 (91.5%)	-

Chest CT scan findings

**Figure 1:**
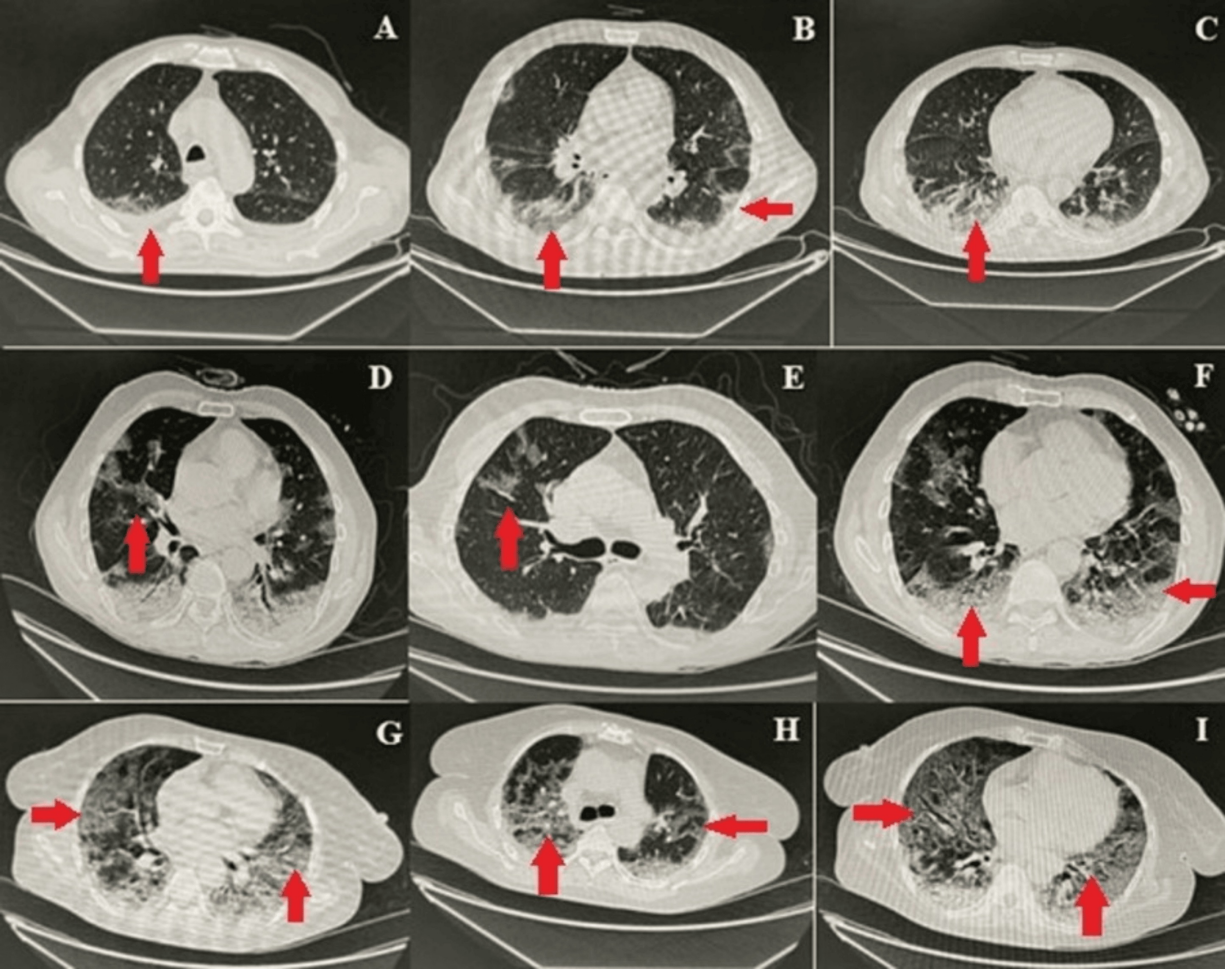
Representative chest CT images showing COVID-19 pneumonia Images A-I show different patterns of opacifications and infiltrates (arrows) ranging from ground glass opacities (e.g., image I) to lobar consolidations (e.g., image D) associated with COVID-19 pneumonia Panels A, B, and C in the figure show COVID-19 pneumonia with pulmonary involvement of 30-40% CO-RADS-6 with chest CTSS-mild. There are bilateral minimal pleural effusion, bilateral peripheral, and basilar ground glass opacities, subpleural reticulations, and consolidations in lower lobes; multiple enlarged mediastinal lymph nodes are seen in the left para-aortic and subcarinal regions. Included sections of the stomach showed circumferential thickening of antrum and pylorus with mural thickness up to 3 cm in the anterior wall. There was about 5-7 ´5.2 cm well-defined soft tissue density lesion inseparable from the tail of the pancreas. Panels D, E, and F show atypical pneumonia COVID-19, CO-RADS-6 with chest CTSS (15/25) (moderate Pulmonary involvement). There are patchy areas of ground glass haze with peripheral predominance in bilateral upper lobes, middle lobe, and lingula. Apical and basal segments of both lower lobes show consolidations with air bronchogram. Panels G, H, and I show atypical pneumonia COVID-19, CO-RADS-5 with radiological chest CTSS (19/25) (severe pulmonary involvement). There are multifocal patches of ground glass haze, and consolidations are seen in both lung fields with peripheral and basal predominance CO-RADS: COVID-19 Reporting and Data System; COVID-19: coronavirus disease 2019; CT: computed tomography; CTSS: computed tomography severity score

Patients in the ICU or isolation ward who had a positive PCR and symptoms of shortness of breath (SOB) and tachypnoea underwent a CT scan to determine the extent of lung involvement. Most of the patients who underwent CT scans had radiologic features consistent with COVID-19 pneumonia, corresponding to a CO-RADS score of 4 (15/25) or 5 (19/25). The most commonly observed findings were basal ground glass haze, opacities, and consolidation (Figure 1). Most of the COVID-19 patients had imaging features suggestive of extensive pulmonary involvement with a chest CTS score of more than 15/25.

Univariate and multivariate logistic regression analyses

Univariable logistic regression analysis revealed a total of 18 possible predictor factors linked to ICU care (p<0.20). Of these, having at least one comorbidity among chronic lung disease, CVD, and diabetes was the factor with the strongest association with ICU admission with univariable OR over 27, followed by renal disease, diarrhea, increased respiratory rate (>24 breaths/minute), and positive RT-PCR with univariable OR between 9 and 15. The remaining eight factors had a weak association with an increased risk of ICU admission; hypokalemia, hyponatremia, and elevated CK had a negative association, whereas elevated urea and ALT, myalgia, and older age had a positive association. The remaining four factors (male sex, presence of cough, presence of fever, and presence of neurological signs) did not show a significant association with the risk of ICU admission (Table 3).

**Table 3:** Risk factor prediction associated with ICU admission with univariable analyses CI: confidence interval; ICU: intensive care unit; OR: odds ratio; RT-PCR: real-time polymerase chain reaction

Parameter	Univariable OR (95% CI)	P-value
Presence of comorbidity	34.44 (18.17, 68.85)	<0.001
Presence of chronic lung disease	34.07 (16.26, 78.81)	<0.001
Presence of cardiovascular disease	29.21 (4.90, 555.55)	<0.001
Presence of diabetes	27.16 (9.87, 95.80)	<0.001
Presence of renal disease	14.10 (1.78, 286.96)	0.02
Presence of diarrhea (vs. normal)	11.44 (3.11, 53.93)	<0.001
Respiratory rate (>24 breaths/minute)	10.49 (5.82, 20.35)	<0.001
Positive RT-PCR (vs. negative)	9.30 (4.07, 26.88)	<0.001
Elevated alanine aminotransferase (vs. normal)	2.41 (1.35, 4.59)	0.004
Presence of myalgia (vs. normal)	2.07 (1.22, 3.65)	0.008
Elevated urea (vs. normal)	1.79 (1.12, 2.92)	0.015
Male sex (vs. female)	1.69 (0.98, 3.08)	0.068
Presence of cough (vs. normal)	1.43 (0.91, 2.27)	0.123
Age, years	1.10 (1.08, 1.12)	<0.001
Presence of fever (vs. normal)	0.68 (0.40, 1.16)	0.145
Hypokalemia (vs. normal)	0.46 (0.20, 0.94)	0.042
Elevated creatinine kinase (vs. normal)	0.41 (0.24, 0.66)	<0.001
Presence of neurological signs	0.41 (0.10, 1.17)	0.172
Hyponatraemia (vs. normal)	0.38 (0.20, 0.67)	0.001

Through a backward stepwise method, a multivariate logical regression model was built with five risk factors and two interaction terms included (Table 4). People with the presence of chronic lung disease and diabetes had greater odds of being admitted into the ICU than those without these comorbidities (OR: 44.55, 95% CI: 14.15, 162.76; OR: 32.74, 95% CI: 7.56, 165.91, respectively, all p<0.001). Compared to females and RT-PCR negativity, males and RT-PCR positivity were negatively associated with ICU inclusion, contrary to previous univariate results, mostly likely due to their significant interactions with age (p<0.001, Table 3). AIC value of 209.7 and the Hosmer-Lemeshow test (ꭓ2=4.42, df=8, p=0.82) suggested a good fit of the model to the data (p=0.96) and the area under the ROC curve (AUC) was 0.951 (95% CI: 0.926, 0.976), indicating that the model had high predictive ability.

**Table 4:** Risk factor prediction associated with ICU admission with multivariable analyses CI: confidence interval; ICU: intensive care unit; OR: odds ratio; RT-PCR: real-time polymerase chain reaction

Parameter	Multivariable OR (95% CI)	P-value
Presence of chronic lung disease	31.98 (10.84, 109.88)	<0.001
Presence of diabetes	24.01 (5.74, 125.79)	<0.001
Positive RT-PCR (vs. negative)	17.18 (5.55, 69.02)	<0.001
Male sex (vs. female)	2.53 (1.14, 5.96)	0.027
Age, years	1.10 (1.08, 1.13)	-

## Discussion

Despite the widespread adoption and roll-out of the global immunization campaign, COVID-19 has still spread since it first appeared in Wuhan, China in December 2019. It is possible that the current immunizations do not offer enough cross-protection against the newer SARS-CoV-2 sub-types: alpha, beta, gamma, delta, and omicron [8].

In resource-limited regions with a high disease burden, medical resources such as CT scans, real-time RT-PCR, and ICU facilities, often fall short of the demand [9]. Hence, basic clinical signs and routine blood tests play a critical role in identifying the severity of COVID-19, particularly regarding ICU admission requirements [10,11]. This association not only aids in efficient resource allocation but also helps frontline healthcare providers make critical decisions regarding patient triage and management [12]. By relying on accessible and widely available clinical markers, this approach ensures that even in resource-constrained clinical settings, patients with severe COVID-19 can be identified promptly and receive appropriate care, potentially reducing the burden on limited ICU resources [13]. It can also significantly contribute to better patient outcomes with more efficient resource utilization [14].

The current study focused on establishing associations between 19 potential clinical and laboratory risk factors and ICU admission in COVID-19 patients. The study identified key risk factors that can be crucial in identifying severe cases requiring ICU care, thus aiding in resource allocation and patient management. Among these risk factors, having at least one comorbidity among chronic lung disease, CVD, and diabetes emerged as the most significant predictor of ICU admission, with a univariable OR exceeding 27. This underscores the importance of considering underlying health conditions when assessing the severity of COVID-19 cases.

Additionally, factors such as renal disease, diarrhea, an increased respiratory rate (>24 breaths/minutes), and a positive RT-PCR test (compared to negative) were also found to have strong associations with ICU admission, with univariable ORs ranging between 9 and 15. To further refine the predictive model, a multivariate logistical regression model was constructed, incorporating five key risk factors. The results indicated a good fit of the model to the data, suggesting that a combination of these risk factors can effectively predict the likelihood of ICU admission among COVID-19 patients. This approach aligns with the broader context of resource constraints and underscores the importance of utilizing readily available clinical and laboratory data to make informed decisions regarding ICU care allocation in regions with limited resources.

Among 500 patients, 90 were transferred to the ICU due to the severity of their disease. The remaining 410 patients were treated in specific COVID-19-designated wards. In these wards, patients received supplemental oxygen when their oxygen saturation was <92%, via a nasal cannula, face mask, or non-breathable mask. In addition to supplemental oxygen, all patients who required oxygen received steroids, antibiotics, and prophylaxis against thromboembolism. However, despite all these measures, mortality in ICU was higher (11/90, 12.22%) compared to COVID ward patients (4/410, 0.97%).

Based on clinical signs and symptoms, the current study found that myalgia was the most common symptom (67.0%). About half had a cough and increased respiratory rate (>24 breaths/minute). Systolic blood pressure (<90 mmHg) and sputum were reported in one-quarter of the patients. In line with our findings, Guan GW et al. and Guan WJ et al. [15,16] reported that fever and cough (88.7% and 67.8%, respectively) were the most frequent characteristics of COVID-19. Our results were also supported by Chan et al. and Wang et al. [10,17]. In addition, our results showed that the only factors significantly associated with the severity of COVID-19 were dyspnea and shortness of breath. In the same way, Zheng et al. showed a strong positive association between dyspnea and shortness of breath with the advancement of COVID-19 to severe and fatal infection.

When it comes to the impact of prevalent CVD and its risk factors, patients with COVID-19 had significantly higher incidence rates for CVD (46, 9.2%), chronic respiratory disease (111, 22.2%), diabetes (27, 5.4%), renal disease, and obesity (103, 20.6%) [18]. These findings were in line with what was reported in a study by (Shi et al., 2020) [19]. Furthermore, a meta-analysis that found similar results suggested that dyspnea be the primary symptom in COVID-19 patients rather than fever as a sign of a bad prognosis [20]. To define the significant clinical and epidemiological characteristics with higher precision, early recognition of risk factors was crucial.

Regardless of age or comorbidities, gender was a risk factor for increased severity but did not correlate with mortality in COVID-19 individuals. This gender resulted in a higher prevalence of male 367 (73.4%) than female 133 (26.6%) among COVID-19 patients, which was associated with general occupational exposure. Another study conducted in the United States, which, although having the largest recorded epidemic of COVID-19 in the world, has only incomplete sex-disaggregated data, because all states and counties do not uniformly submit this data to the Centre for Disease Control and Prevention [16]. Men may have a greater frequency due to a higher prevalence of pre-existing disorders (CVD, chronic lung disease, diabetes, hypertension, and obesity), as well as higher risk behaviors (smoking and other occupational exposures) [21].

The sociodemographic setting in Pakistan is another significant determinant. Men are more likely than women to interact with others in the community (e.g., at supermarkets, places of employment, and job searches), and as a result, they are more likely to contract the coronavirus. Given the high rates of COVID-19 transmission and mortality, it is imperative to obtain a reliable and early diagnosis of the disease [22]. We used real-time RT-PCR analyses to verify viral presence to provide an early and precise diagnosis. Applying this technique has several disadvantages, including a large number of samples, a small number of personnel skilled in carrying out the tests listed above, and inadequate lab capacity; as a result, results may take longer to arrive. It is essential to find additional biomarkers that are rapidly quantifiable.

Simple, affordable, and readily available are hematological and biochemical tests (strongly correlated with PCR results; r=0.963, p<0.01). Hematologic and serum biochemical abnormalities in patients with mild, moderate, and severe disease may be well categorized in this clinical investigation, potentially allowing for significant discrimination to be included in risk stratification models for more realistic triaging. More often than patients with milder disease, patients with severe COVID-19 seem to have indications of liver dysfunction. As with many other biochemical indicators, an elevation in ALT [23], AST [24], and a decrease in total protein levels may be useful for monitoring patients admitted to the ICU. Our results are aligned with Zhang et al.'s study [25]. Similar findings have been reported by other researchers as well [26]. 

Moreover, acute-phase reactant production is induced by viral infection [9]. When comparing individuals with severe COVID-19 to those with mild to moderate COVID-19, our results revealed considerably higher levels of acute phase reactants (CRP and ferritin) as well as LDH. As the condition worsens and moves from a mild to a moderate or severe state, these biochemical markers rise. This data may be utilized in risk stratification for predicting severe and fatal COVID-19 in hospitalized patients. Numerous inflammatory disorders result in a rise in CRP. Those with COVID-19 infection have higher rates of it (75-293%), especially in cases of severe illness [27]. These findings were similar to most of the studies conducted by Liu et al. and Rodriguez et al. [28,29]

Furthermore, we analyzed the hematological indices of the COVID-19 patients who were included; these indices are crucial since they give clinicians helpful indicators of prognosis. This indicated that patients with severe infection had considerably lower mean values of white blood cell and lymphocyte counts than patients with mild to moderate COVID-19 infection. Furthermore, there was a significant association found between lymphopenia and the severity of COVID-19. The majority of COVID-19 patients had leukopenia and lymphopenia upon admission, according to Fan et al. [30]. The majority of COVID-19 patients had leukopenia and lymphopenia upon admission, according to Fan et al. As shown in Table 4, other blood components such as neutrophils and platelets were only somewhat predictive in differentiating between mild and severe COVID-19. A component of the hyper-inflammatory state, neutrophilia has a significant, harmful role in COVID-19 and associated diseases [31]. Our study aligns with Cheng [32] and Huang et al. [33]. Thrombocyte and lymphocyte numbers frequently decline due to viral pneumonia's effects on the immune system. It is also possible that these outcomes were impacted by additional variables like comorbidities, anemia, and lifestyle choices like smoking cigarettes [34].

Patients with a suspected SARS-CoV-2 infection were able to be separated and treated in time for recovery because of the pathological lesions shown by CT scans used to diagnose viral pneumonia, which improved patient care [2,35]. The findings of the nucleic acid test revealed the diagnosis of viral pneumonia 3.0 days before the CT scan. In addition, we have confidence that a CT scan has high accuracy and may be helpful as a standard approach for the diagnosis of COVID-19, as the timing of testing about the progression of the disease can influence the accuracy of the test results. According to research by Jiang and Pan, this result is consistent. [36,37]. CT scan is still limited in its ability to identify certain viruses, though. Recognizing that the CT findings of COVID-19 differ and sometimes overlap with viruses from the same family, including SARS-CoV-1 and MERS-CoV, as well as with infections caused by viruses from other families, like adenovirus, is important for radiologists [38].

According to the current study's findings, the left para-aortic and sub-carinal regions exhibit multiple enlarged mediastinal lymph nodes, bilateral minimal pleural effusion, bilateral peripheral and basilar ground glass opacities, and sub-pleural reticulations and consolidations in the lower lobes. In both lung regions, there are several multi-located patches of ground glass haze and consolidations, with a peripheral predominance in the bilateral upper lobes, middle lobes, and lingual areas [18,39]. The current study's findings demonstrated that the imaging characteristics of COVID-19 pneumonia are typical, with the mid-to-lower zones being more impacted and bilateral multilobar ground-glass opacities with the peripheral distribution. No pulmonary nodules, mediastinal lymphadenopathy, or pleural effusion was visible on either side. These results aligned with those presented by Saqlain [22].

In the present study, patients with greater lung consolidation and older ages showed higher death rates. According to recent findings (145), older patients (≥65 years old) had a higher likelihood of having a severe COVID-19 illness. The clinical classification of severity indicated that men tended to acquire more serious cases than women [20,40]. Furthermore, we discovered that the proportion of elderly (≥65 years) patients was much higher in the dead than in the survivors (60% in nine deceased patients vs. 29% in 145 survivors). Men were more likely to die from COVID-19, even though both sexes were equally susceptible. These outcomes are comparable to Gonzalez's findings [41,42]. Male and female patient ages were similar in both the deceased and the survivors of COVID-19, even though the deceased patients were substantially older than the survivors. Age had a significant association with patients (p<0.05) [43].

Limitations

This study has a few limitations, even though it provides insightful information about clinical and laboratory risk factors for ICU admission in COVID-19 patients. Firstly, selection bias may have been introduced due to the retrospective design, which also restricts the capacity to determine causal correlations. Moreover, the research was carried out in a particular area and for a specified duration, which could restrict the applicability of the results to different demographics or stages of the crisis. Furthermore, the wide variety of patient characteristics and treatment modalities could not be adequately represented by relying merely on data from two institutions. Moreover, by concentrating on quantifiable risk variables, the study might have neglected the impact of other contextual factors and subjective clinical judgments on triage choices.

## Conclusions

The study found that several factors, including advanced age; male gender; and comorbid conditions like diabetes, chronic lung disease, and CVD had a strong association with ICU admission among COVID-19 patients. Similarly, clinical features such as tachypnea and diarrhea, and biochemical markers like elevated CK were also associated with increased odds of ICU admission. These clinical markers can be used for early risk stratification of COVID-19 patients in resource-limited settings, thereby improving healthcare efficiency and as well as clinical outcomes.
